# Mepazine Inhibits RANK-Induced Osteoclastogenesis Independent of Its MALT1 Inhibitory Function

**DOI:** 10.3390/molecules23123144

**Published:** 2018-11-30

**Authors:** Laura Meloni, Lynn Verstrepen, Marja Kreike, Jens Staal, Yasmine Driege, Inna S. Afonina, Rudi Beyaert

**Affiliations:** 1 Unit of Molecular Signal Transduction in Inflammation, VIB-UGent Center for Inflammation Research, VIB, 9052 Ghent, Belgium; laurameloni86@gmail.com (L.M.); lynn.verstrepen@prodigest.eu (L.V.); marja.kreike@irc.vib-ugent.be (M.K.); yasmine.driege@irc.vib-ugent.be (Y.D.); inna.afonina@irc.vib-ugent.be (I.S.A.); 2 Department of Biomedical Molecular Biology, Ghent University, 9052 Ghent, Belgium

**Keywords:** osteoclastogenesis, mepazine, MALT1, RANK, NF-κB, phenothiazine, paracaspase, osteoclast

## Abstract

Mucosa-associated lymphoid tissue lymphoma translocation protein 1 (MALT1) is an intracellular cysteine protease (paracaspase) that plays an integral role in innate and adaptive immunity. The phenothiazine mepazine has been shown to inhibit the proteolytic activity of MALT1 and is frequently used to study its biological role. MALT1 has recently been suggested as a therapeutic target in rheumatoid arthritis. Here, we analyzed the effect of mepazine on the receptor activator of nuclear factor κ-B (RANK)-induced osteoclastogenesis. The treatment of mouse bone marrow precursor cells with mepazine strongly inhibited the RANK ligand (RANKL)-induced formation of osteoclasts, as well as the expression of several osteoclast markers, such as TRAP, cathepsin K, and calcitonin. However, RANKL induced osteoclastogenesis equally well in bone marrow cells derived from wild-type and *Malt1* knock-out mice. Furthermore, the protective effect of mepazine was not affected by MALT1 deficiency. Additionally, the absence of MALT1 did not affect RANK-induced nuclear factor κB (NF-κB) and activator protein 1 (AP-1) activation. Overall, these studies demonstrate that MALT1 is not essential for RANK-induced osteoclastogenesis, and implicate a MALT1-independent mechanism of action of mepazine that should be taken into account in future studies using this compound.

## 1. Introduction

Phenothiazines are a family of chemical compounds characterized by a common tricyclic phenothiazine ring and a variable side chain. The nature of the side chain specifies different derivatives and determines their inhibitory potential toward various substrates [1]. Phenothiazines are best known from their long history as neuroleptic antipsychotic drugs due to their dopamine blocking properties [2]. Specific phenothiazines, including mepazine, were found to inhibit mucosa-associated lymphoid tissue lymphoma translocation 1 (MALT1) [3,4] and to exert therapeutic activity in preclinical models of several diseases, including multiple sclerosis [5], activated B cell subtype of diffuse-large B cell lymphoma (ABC-DLBCL) [3], viral infection [6,7], and colitis [8]. MALT1 (PCASP1 [9]) is an intracellular signaling protein that plays a key role in innate and adaptive immunity [10,11]. More specifically, MALT1 acts as a scaffold protein for downstream signaling proteins leading to NF-κB activation. In addition, MALT1 protease activity further fine-tunes gene expression by cleaving a number of substrates [10,11]. Its function is best known in the context of T cell receptor-induced signaling leading to proliferation, survival, and activation of T lymphocytes, but MALT1 also plays an important role in several other cell types, including myeloid cells and nonimmune cells [11]. We were interested in a potential role of MALT1 in the formation of osteoclasts from bone marrow precursor cells. Several phenothiazines were described to inhibit osteoclastic bone resorption in vivo and in vitro [12,13,14,15,16,17,18,19]. Mechanistically, the phenothiazines chlorpromazine, promethazine, and trifluoperazine were shown to inhibit the receptor activator of nuclear factor κB (RANK)-induced osteoclast differentiation from bone-marrow cells (BMCs) [19]. Mature osteoclasts are multinucleated giant bone resorbing-cells with the ability to degrade the mineralized matrices of bone and calcified cartilage [20]. Osteoclasts differentiate from hematopoietic stem cells and express specific differentiation markers: Tartrate-resistant acid phosphatase (TRAP), matrix metalloproteinase-9, cathepsin-K, carbonic anhydrase II, osteopetrosis-associated transmembrane protein 1, vacuolar type ATPase containing the a3 subunit, chloride channel, and the calcitonin receptor, both during development and as mature cells [20]. Macrophage-colony stimulating factor (M-CSF) and RANK ligand (RANKL) are two indispensable factors for osteoclastogenesis [21], and their deficiency in mice leads to a complete lack of osteoclasts [22]. In particular, M-CSF is necessary for the survival and the proliferation of preosteoclasts, while RANKL is required for their final differentiation. RANKL signaling results in the activation of different transcription factors, including nuclear factor κB (NF-κB) [23] and activator protein 1 (AP-1) [24]. In addition, RANKL potently induces the expression and activation of the nuclear factor of activated T cells cytoplasmic 1 (NFATc1), a master regulator of osteoclast differentiation [25]. Of interest, the MALT1 substrates A20 [26], CYLD [27], and RelB [28] have previously been associated with osteoclastogenesis. More specifically, CYLD was found to inhibit RANK-induced signaling by deubiquitinating TRAF6, and its physiological importance is reflected by the fact that CYLD-deficient mice develop osteoporosis, due to accelerated osteoclastogenesis [29]. In addition, mice with an A20 deficiency in myeloid cells showed enhanced osteoclastogenesis associated with the development of severe erosive polyarthritis [30]. Finally, RelB-mediated noncanonical NF-κB activation is required for full RANKL-induced osteoclast maturation [31]. The reported anti-osteoclastogenic effect of phenothiazines structurally related to the MALT1 inhibitor mepazine, as well as the function of some MALT1 substrates in osteoclastogenesis, led us to hypothesize a novel role for MALT1 in osteoclastogenesis. We therefore tested the effect of pharmacological and genetic MALT1 inhibition on RANK-induced osteoclastogenesis in vitro. We show that treatment of BMCs with mepazine completely inhibits RANK-induced osteoclastogenesis. However, we also found that mepazine is equally potent in the absence of MALT1, and that MALT1 deficiency does not affect RANK-induced signaling and osteoclastogenesis. These results exclude a role for MALT1 in RANK-induced osteoclastogenesis and implicate a MALT1-independent mechanism of action of mepazine that should be taken into account in future studies using this compound.

## 2. Results

### 2.1. Mepazine Inhibits RANK-Induced Osteoclastogenesis Independent of MALT1

**Figure 1:**
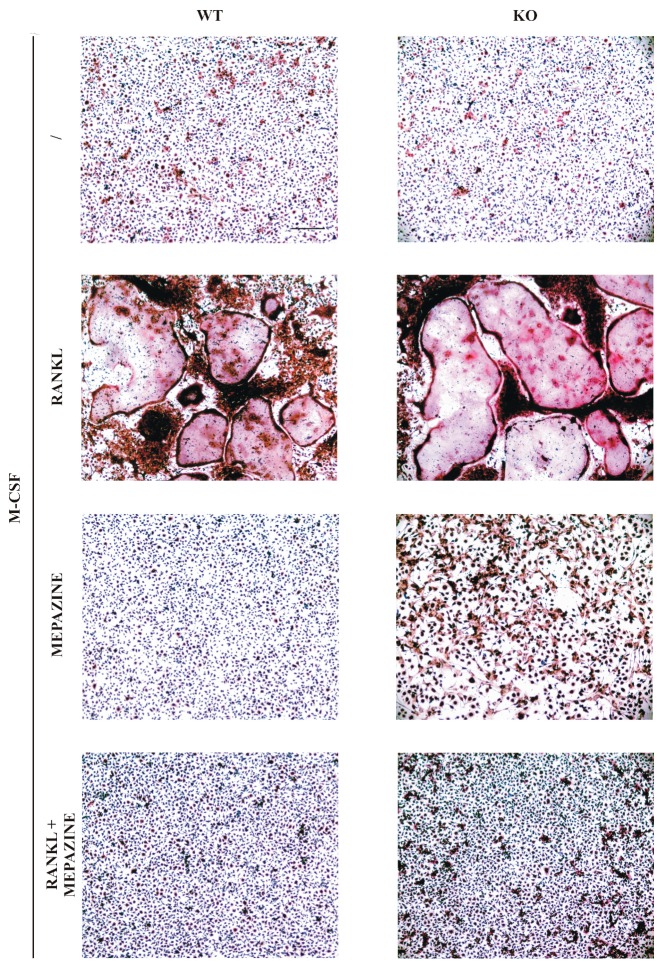
Mepazine inhibits RANK ligand (RANKL)-induced osteoclastogenesis independently of mucosa-associated lymphoid tissue lymphoma translocation (MALT1). Bone marrow cells (BMCs) isolated from wild-type (WT) and *Malt1* knock-out (KO) mice were stimulated every two days with macrophage-colony stimulating factor (M-CSF) (20 ng/mL) plus RANKL (50 ng/mL) in the presence or absence of mepazine (13 µM). Samples without mepazine were treated with an equal volume (0.1% final concentration) of DMSO as solvent control. The / symbol represents that no RANKL or mepazine was added. At day 9, cells were fixed and stained for tartrate-resistant acid phosphatase (TRAP). Microscopic analysis shows the appearance of very large multinucleated TRAP-positive cells (=typical for osteoclasts) after M-CSF plus RANKL treatment. Scale bar represents 100 µm. Results are representative of three independent experiments.

Several phenothiazines were shown to inhibit RANK-induced osteoclast differentiation from BMCs [19]. To test if mepazine, a phenothiazine shown to inhibit MALT1 [3], has a similar anti–osteoclastogenic effect, we treated BMCs with M-CSF and RANKL in the presence or absence of mepazine. The mepazine concentration used was equal to the concentration that shows efficient inhibition of MALT1 protease activity in stimulated T cells [5]. At day 9, when osteoclast formation was microscopically visible in M-CSF plus RANKL-treated cells, cells were stained for the osteoclast marker TRAP. As shown in Figure 1, mepazine completely prevented RANKL-induced osteoclast differentiation, suggesting a potential novel role of MALT1 in osteoclastogenesis. However, in the same experiment, we also treated BMCs from both *Malt1*^+/+^ and *Malt1*^−/−^ mice with M-CSF and RANKL, and found osteoclast formation to be equally induced in both wild-type and knock-out cells (Figure 1). These data illustrate that MALT1 is dispensable for RANKL-induced osteoclastogenesis, which also led us to question the MALT1-dependency of the protective effect observed with mepazine. Indeed, RANKL-induced osteoclast formation was equally inhibited by mepazine in *Malt1*^+/+^ and *Malt1*^−/−^ cells (Figure 1), demonstrating that mepazine exerts an anti-osteoclastogenic effect independent of its MALT1 inhibitory capacity.

**Figure 2:**
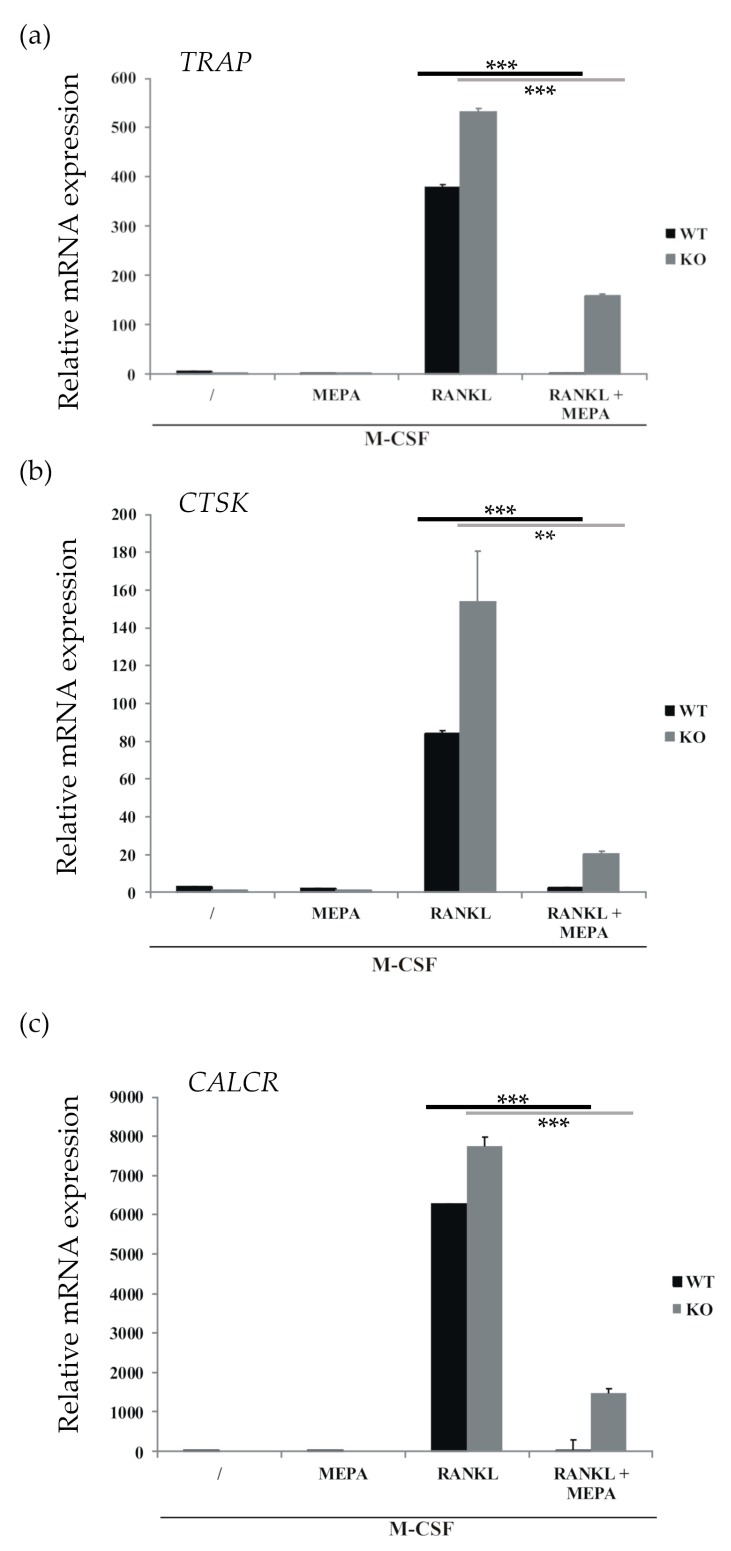
MALT1-independent inhibition of osteoclast-specific gene induction by mepazine. BMCs isolated from *Malt1* wild-type (WT) and *Malt1* knock-out (KO) mice were differentiated into osteoclasts by treatment with M-CSF (20 ng/mL), as well as RANKL (50 ng/mL) in the presence or absence of mepazine (MEPA, 13 µM) every two days. Samples without mepazine were treated with an equal volume (0.1% final concentration) DMSO as solvent control. The / symbol represents that no RANKL or mepazine was added. mRNA was extracted at day 9 and qPCR was performed for (**a**) *TRAP*, (**b**) *CTSK* (Cathepsin K) and (**c**) *CALCR* (Calcitonin receptor). Values are the mean of technical triplicates ± S.D. Data are representative of two independent experiments. Statistical differences were determined by Student’s *t*-test, ** represents *p* ≤ 0.01 and *** represents *p* ≤ 0.001.

To further exclude the role of MALT1 in the inhibitory effect of mepazine on osteoclast development, RANKL-induced osteoclast-specific gene expression in *Malt1* wild-type and MALT1-deficient cells was tested in the presence or absence of mepazine. As shown in Figure 2, RANKL clearly upregulated osteoclast-specific genes, like *TRAP*, *CTSK* (cathepsin K), and *CALCR* (calcitonin receptor), both in wild-type and MALT1-deficient cells, highlighting that MALT1 is dispensable for RANK-induced osteoclastogenesis. Of note, absence of MALT1 even slightly increased RANK-induced gene expression for reasons that are still unclear. Most importantly, RANK-induced gene expression was strongly reduced by mepazine in both *Malt1*^+/+^ and *Malt1*^−/−^ cells, further demonstrating that the inhibitory effect of mepazine on osteoclast formation is MALT1-independent.

### 2.2. MALT1 Is not Involved in RANK-Induced NF-κB and AP1 Signaling

**Figure 3:**
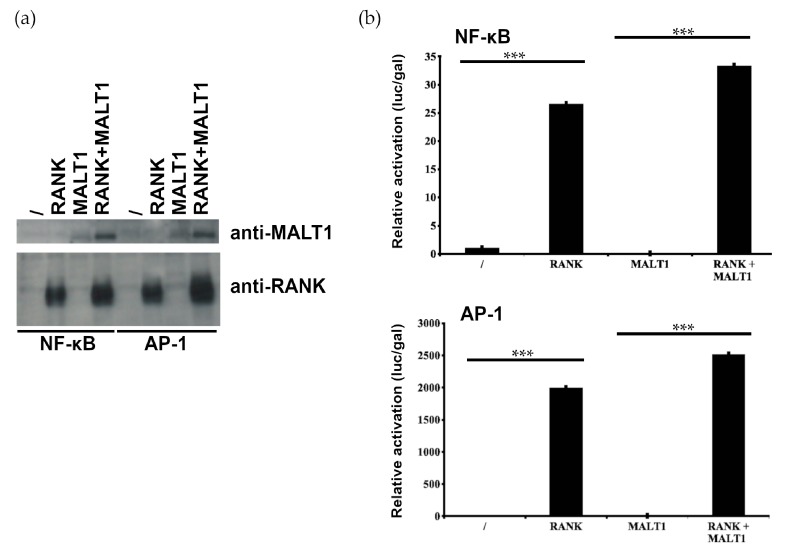
MALT1 is not necessary for RANK-induced NF-ĸB and AP-1 signaling. MALT1-deficient HEK293T cells or cells reconstituted with MALT1 (as indicated on the X-axis) were transiently transfected with a RANK expression plasmid, a plasmid constitutively expressing β-galactosidase, and plasmids expressing either an NF-κB-dependent or AP-1-dependent luciferase reporter gene. The / represents set-ups where neither RANK nor MALT1 is expressed. Statistical differences were determined by Student’s *t*-test, *** represents *p* ≤ 0.001. (**a**) Expression of MALT1 and RANK was verified by Western blotting. The four left lanes correspond to the samples analyzed in (**b**) for NF-κB reporter activation, the last four lanes correspond to the samples analyzed in (**b**) for AP-1 reporter activation. (**b**) Luciferase activity was measured 24 h after transfection and normalized to β-galactosidase expression (plotted as luc/gal). Values are the mean of triplicates ± S.D. Data shown are representative of two independent experiments.

Engagement of RANKL with its receptor RANK induces NF-κB and AP-1 activation, which plays an important role in osteoclastogenesis [32,33]. To analyze the role of MALT1 in RANK-induced NF-κB and AP-1 signaling, we transfected MALT1-deficient HEK293T cells with RANK, whose overexpression is known to activate downstream signaling in a ligand-independent manner [34,35], and either a NF-κB-dependent or AP-1-dependent luciferase reporter plasmid. We similarly analyzed the effect in MALT1-deficient HEK293T cells that were reconstituted with MALT1. Expression levels of transfected RANK and MALT1 were checked via western blotting (Figure 3a). RANK overexpression reproducibly increased the expression of co-transfected MALT1, most likely reflecting effects of RANK signaling on the activity of the cytomeglavirus (CMV) promoter that drives MALT1 expression [36,37]. Most importantly, RANK overexpression activated NF-κB and AP-1-dependent reporter gene expression equally well in MALT1-deficient and MALT1-expressing cells (Figure 3b), demonstrating that NF-κB and AP-1 signaling in response to RANK activation is MALT1-independent. These results are consistent with our observation that RANKL-induced osteoclastogenesis is MALT1-independent, and further support our conclusion that mepazine inhibits RANK-induced osteoclastogenesis independent of its MALT1 inhibitory activities.

### 2.3. Mepazine Inhibits CaMKII Phosphorylation and NFATc1 Expression Independently of MALT1

**Figure 4:**
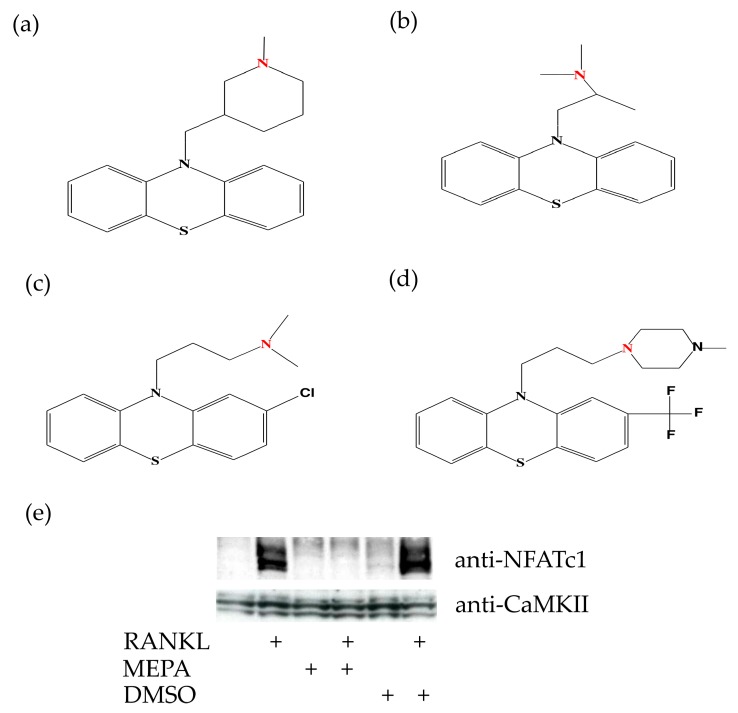
Mepazine inhibits RANKL-induced NF-AT1 expression. (**a**) Molecular structure of Mepazine (**b**) Promethazine (**c**) Chlorpromazine (**d**) Trifluoperazine, with the critical N group proposed to influence calmodulin highlighted in red. (**e**) As in Figure 1 and Figure 2, BMCs were stimulated for 9 days with M-CSF (20 ng/mL) plus RANKL (50 ng/mL) in the presence or absence of mepazine (MEPA; 13 µM) or DMSO (0.1% final concentration) as solvent control every two days. Cell lysates were analyzed by Western blot for NFATc1 expression. CaMKII expression is used as loading control.

Besides NF-κB signaling, the transcription factor NFATc1 also plays an integral role in the RANKL-induced transcriptional program during the terminal differentiation of osteoclasts. NFATc1 is strongly induced and activated by RANKL via a mechanism that is dependent on the Ca^2+^/calmodulin-regulated phosphatase, calcineurin [25]. The chemical structure of mepazine contains two hydrophobic groups and an N group (Figure 4a), which are the essential structural elements in other phenothiazines (for example, Promethazine, Chlorpromazine, and Trifluoperazine (Figure 4b–d)) that were previously shown to inhibit calmodulin activity [38]. Earlier studies suggest that the critical N group is positively charged by binding a proton under physiological pH, and that this contributes to electrostatic interactions with the negatively charged calmodulin [38]. It is therefore likely that mepazine affects osteoclast differentiation by inhibiting the calmodulin-dependent expression and activation of NFAT. Indeed, mepazine completely prevented RANK-induced NFATc1 expression in M-CSF treated BMCs (Figure 4e), indicating that mepazine may exert its anti-osteoclastogenic function by inhibiting calmodulin-dependent NF-AT1 expression and activation.

## 3. Discussion

In the present study, we show that the MALT1 inhibitor mepazine strongly inhibits RANK-induced osteoclastogenesis via a MALT1-independent mechanism. Mepazine has been frequently used in several in vitro and in vivo studies to demonstrate a role for MALT1 [3,4,5,6,7,8,39,40,41,42,43,44]. Most importantly, the therapeutic effect of mepazine in mouse models of autoimmune disease and ABC-type DLBCL has strengthened the belief in therapeutic targeting of MALT1. The rationale for MALT1 as a therapeutic target is based on much more evidence than the inhibitory effect of mepazine, including supportive evidence by other MALT1 inhibitors and genetic inactivation of MALT1 protease activity in mouse models [11]. However, it cannot be excluded that the observed therapeutic effects of mepazine in these previous studies also partially reflect some of its MALT1-independent activities. Care should therefore be taken in the future when interpreting results obtained with this inhibitor. A recent report suggested inhibition of osteoclastogenesis and protection in a model of rheumatoid arthritis by the structurally unrelated MALT1 active site inhibitor MI-2 [45]. However, this study did not control for a possible role of off-target activities of MI-2, which was recently shown to be a very nonspecific inhibitor [46]. This highlights the need to verify on-target effects of an inhibitor when used for studying the biological role of its presumed target, preferably by also doing inhibitor treatments of a genetic knock-out of the intended target protein [47].

Independent of the involvement of MALT1, the clear protective effect of mepazine on RANK-induced osteoclastogenesis is of significant therapeutic interest because of its previous clinical use as an antipsychotic drug [2]. More specifically, mepazine was commercialized in the late 1950s under the name Pacatal and administered as a tranquilizer to treat anxiety, aggression, and impulsiveness [48]. It was subsequently removed from the market in the late 1960s because its efficacy was not significant or close to zero compared to other available antipsychotic drugs [49]. Moreover, it presented some side effects, such as granulocytopenia, hypotension, urinary retention, and paralytic ileus [50]. Nevertheless, because of its MALT1 inhibitory effects, a repurposing of mepazine for the treatment of autoimmunity and ABC-DLBCL has been proposed [4]. Our present observation that mepazine inhibits osteoclastogenesis via a different pathway further strengthens the therapeutic potential of mepazine. In fact, a recent patent application reports that mepazine treatment protects mice from the development of arthritis in a collagen-induced arthritis model [51]. As a matter of fact, it is shown that treatment with mepazine leads to a reduction in pannus formation and bone resorption, indicating a decrease in the number of osteoclasts, which is in line with our in vitro results.

Our observation that RANK-induced osteoclastogenesis proceeds equally well in wild-type versus *Malt1* knock-out cells allows us to conclude that MALT1 is dispensable for RANK-induced osteoclastogenesis in vitro. Nevertheless, these results do not exclude a role for MALT1 in osteoclastogenesis via other RANK-independent mechanisms. In this context, MALT1 has been linked to signaling downstream of the ITAM-containing adaptor proteins DAP12 and FcγR [52], which cooperate with the immunoreceptors TREM-2 and OSCAR as co-stimulators of RANK-induced osteoclastogenesis [53,54]. Lately, it has been shown that DAP12 stimulation through IL-23, a cytokine belonging to the IL-12 family, is able to induce osteoclastogenesis through the activation of NFATc1 [55]. In addition, desialylated immunoglobulin G (IgG) immune complexes were recently shown to increase osteoclastogenesis in vivo and in vitro via binding to FcγRII and FcγRIII, but not FcγRI [56]. Therefore, it could be of interest to evaluate a possible difference in the number of osteoclasts in *Malt1*^+/+^ and *Malt1*^−/−^ mice by ex vivo TRAP staining on bone slices or by measuring bone density using a CT scan. It should be mentioned, however, that *Malt1*^−/−^ mice suffer from Treg deficiency, which is known to contribute to increased osteoclastogenesis and decreased bone density [57]. This implies that one can expect *Malt1*^−/−^ mice to have a lower bone density, independent from a direct role of MALT1 in osteoclastogenesis. Final proof for a direct role of MALT1 in osteoclastogenesis would therefore need the use of osteoclast-specific *Malt1*^−/−^ mice. Of interest, MALT1 deficiency in patients has been associated with combined immunodeficiency, and a case of human MALT1 deficiency in a 15-year-old female was shown to experience significant growth delay with short stature, low weight, and delayed bone age [58]. Moreover, she had very low bone mineral density and fractured her femur and both tibiae after low-impact injuries, which were reversed after transplantation of healthy hemapoetic stem cells [58,59].

It will be of interest to identify the real target of mepazine that is responsible for its protective effect against RANK-induced osteoclastogenesis. Our results indicate that mepazine is influencing the Ca^2+^-dependent signaling pathway for osteoclastogenesis, possibly via a direct effect on calmodulin. It is worth mentioning that the Ca^2+^-calmodulin-calcineurin pathway is also involved in T cell receptor (TCR) signaling. For example, CaMKII is able to phosphorylate BCL10 [60,61] and CARMA1 [62], while calcineurin is able to dephosphorylate BCL10 [63], two components of the CBM complex, where MALT1 represents the third component. Therefore, it is not unlikely that some of the reported effects of mepazine on TCR-induced immune responses reflect not only its effect on MALT1, but also on calcineurin. In this context, it is worth mentioning that the calcineurin inhibitor cyclosporine A is well known because of its immunosuppressive activities [64]. The field is clearly in need for more specific small compound inhibitors of MALT1, and several new developments seem to go into this direction [46].

## 4. Materials and Methods

### 4.1. Mice

*Malt1*^−/−^ mice [65], backcrossed to C57BL/6 mice, were kindly provided by Dr. Tak W. Mak (Ontario Cancer Institute, Toronto, ON, Canada). *Malt1*^−/+^ mice were intercrossed to generate *Malt1*^+/+^, *Malt1*^−/+^, and *Malt1*^−/−^ offspring. Mice were housed in individually ventilated cages in a specific pathogen-free animal facility. Mice were supplied with water and food *ad libitum.* Euthanasia was performed in compliance with the local guidelines of the University of Ghent Ethics Committee. All experiments were performed ex vivo on cells isolated from euthanized mice. Both male and female mice were used at an age of 8 to 12 weeks.

### 4.2. Osteoclast Cell Culture and TRAP Staining

Mouse BMCs were obtained from tibiae as previously described [66] and 2 × 10^6^ cells were seeded and cultured for 10 days in 6-well dishes in α-MEM (Minimal Essential Medium Alpha, Thermo Fisher Scientific, Waltham, MA, USA) with 10% fetal bovine serum (FBS) (Thermo Fisher Scientific, Waltham, MA, USA), 100 IU/mL penicillin and 100 μg/mL streptomycin (Thermo Fisher Scientific, Waltham, MA, USA). Cells were stimulated every 2 days with 20 ng/mL M-CSF (Protein Service Facility, VIB, Ghent, Belgium) and 50 ng/mL RANKL (Bio-Techne, R&D Systems, Minneapolis, MN, USA) to obtain osteoclasts. Mepazine acetate (kindly provided by Dr. Krappmann) was dissolved in DMSO and added to the cells at a concentration of 4.5 μg/mL (13 µM) every 2 days. The Mepazine concentration was chosen based on what we previously had found an efficient dose to block MALT1 protease activity in stimulated T cells [5]. On day 9, osteoclasts were stained for TRAP using the Acid Phosphatase, Leukocyte (TRAP) kit according to manufacturer’s instructions (Sigma-Aldrich Corporation, Saint Louis, MO, USA). Pictures were taken with an Olympus BX51 discussion microscope (Olympus, Tokyo, Japan) with 10X dry objective (0.25 NA).

### 4.3. Quantitative Real-Time Polymerase Chain Reaction (qRT-PCR)

On day 9, 500 μL TRIzol reagent (ThermoFisher Scientific) was added to the cells and total RNA was isolated using the Aurum Total RNA Isolation Mini Kit according to the manufacturer’s instructions (Bio-Rad, Hercules, CA, USA). cDNA was prepared from 1 μg of total RNA using the iScript cDNA synthesis kit according to the manufacturer’s instructions (Bio-Rad, Hercules, CA, USA). qPCR was performed with SensiFAST^TM^ Sybr No-ROX kit (Bioline, London, UK) and specific primers on a LightCycler 480 (Roche, Basel, Switzerland). Real-time PCR reactions were performed in triplicates and analyzed via the qBASE program (Biogazelle, Ghent, Belgium). mRNA expression of the genes of interest, measured by qPCR, was normalized by the program to the expression of the housekeeping genes, and the error bars on the graphs represent the standard error of the mean (SEM) of three technical replicates. The following specific forward (FW) and reverse (REV) primers were used:


**ACTIN FW**
GCTTCTAGGCGGACTGTTACTGA
**MALT1 FW**
GGACAAAGTCGCCCTTTTGAT
**ACTIN REV**
GCCATGCCAATGTTGTCTCTTAT
**MALT1 REV**
TCCACAGCGTTACACATCTCA
**GADPH FW**
TGAAGCAGGCATCTGAGGG
**TRAP FW**
TGGTCCAGGAGCTTAACTGC
**GADPH REV**
CGAAGGTGGAAGAGTGGGAG
**TRAP REV**
GTCAGGAGTGGGAGCCATATG
**CATHEPSIN K FW**
GTGGGTGTTCAAGTTTCTGC
**CALCITONIN receptor FW**
CTCCAACAAGGTGCTTGGGA
**CATHEPSIN K REV**
GGTGAGTCTTCTTCCATAGC
**CALCITONIN receptor REV**
GAAGCAGTAGATAGTCGCCA

### 4.4. Luciferase Assay and Western Blot Analysis

MALT1-deficient HEK293T cells (clone #36), which have been used to determine MALT1-dependent pathways in several previous studies [9,41,67], were cultured in Dulbecco’s modified Eagle’s medium (DMEM), supplemented with 10% fetal calf serum and 2 mM l-glutamine. For luciferase assays, 50,000 MALT1 deficient HEK293T cells were seeded, and the day after, transiently transfected by the calcium phosphate precipitation method with specific expression plasmids, NF-ĸB- or AP-1-dependent luciferase reporter plasmids, and a constitutively expressed β−galactosidase reporter plasmid, as indicated. All plasmids were obtained from the BCCM/GeneCorner (www.genecorner.ugent.be) plasmid collection, Ghent, Belgium: pMX-RANK (LMBP 8952), pCD-MK (MALT1) (LMBP 5536), pNFconLuc (LMBP 3248), 3× AP1 pGL3 (LMBP 8820), pACTβGal (LMBP 4341). 24 h after transfection, cells were lysed in 200 μL lysis buffer (25 mM Tris-phosphate pH 7.8, 2 mM dithiothreitol, 2 mM 1,2-cyclohexanediaminetetraacetic acid, 10% glycerol and 1% Triton X-100). Luciferase activity was measured in the Glomax luminometer (Promega, Madison, WI, USA) upon addition of substrate buffer to a final concentration of 470 μM luciferin, 270 μM co-enzyme A and 530 μM ATP. β-Galactosidase activity was assayed with chlorophenol red β-galactopyranoside substrate (Roche Molecular Biochemicals, Vilvoorde, Belgium) and the absorbance was measured with a Benchmark microplate reader (iMark, Bio-Rad, Hercules, CA, USA). NF-ĸB and AP-1-dependent expression of luciferase are presented relative to the constitutive expression of β-galactosidase to normalize for potential differences in transfection efficiency.

For Western blot analysis, cells were lysed in Laemmli buffer, heated to 95 °C, loaded onto an SDS-polyacrylamide gel and transferred to a nitrocellulose blot by semi-dry immunoblotting. Blots were incubated with anti-RANK (sc-374360, Santa Cruz Biotechnology, Dallas, TX, USA), anti-MALT1 (sc-46677, Santa Cruz Biotechnology, Dallas, TX, USA), NFAT1c (556602, BD Pharmingen, Erembodegem, Belgium) or CaMKII (3362, Cell Signaling Technology Inc., Leiden, The Netherlands) primary antibodies, and horse radish peroxidase (HRP)-conjugated anti-rabbit or anti-mouse IgG secondary antibodies (GE Healthcare Life Sciences, Diegem, Belgium), followed by detection via enhanced chemiluminescence.

### 4.5. Chemical Drawings

Chemical structures were drawn with Chemtool (http://ruby.chemie.uni-freiburg.de/~martin/chemtool/chemtool.html) [68] and further adjusted in Inkscape (www.inkscape.org).
